# Remotely monitored physical activity from older people with cardiac devices associates with physical functioning

**DOI:** 10.1186/s12877-024-05083-1

**Published:** 2024-06-17

**Authors:** J.K. Taylor, N. Peek, A.S. Greenstein, C. Sammut-Powell, G.P. Martin, F.Z. Ahmed

**Affiliations:** 1 Centre for Health Informatics, Division of Informatics, Imaging and Data Sciences, University of Manchester, Oxford Road, Manchester, M13 9P UK; 2 Department of Cardiology, Manchester University Hospitals NHS Foundation Trust, Oxford Rd, Manchester, UK; 3 THIS Institute (The Healthcare Improvement Studies Institute), University of Cambridge, Cambridge, UK; 4 Division of Cardiovascular Sciences, Faculty of Biology, Medicine and Health, University of Manchester, Manchester, UK

**Keywords:** Cardiac devices, Remote monitoring, Physical activity, Frailty, Physical functioning

## Abstract

**Introduction:**

Accelerometer-derived physical activity (PA) from cardiac devices are available via remote monitoring platforms yet rarely reviewed in clinical practice. We aimed to investigate the association between PA and clinical measures of frailty and physical functioning.

**Methods:**

The PATTErn study (A study of Physical Activity paTTerns and major health Events in older people with implantable cardiac devices) enrolled participants aged 60 + undergoing remote cardiac monitoring. Frailty was measured using the Fried criteria and gait speed (m/s), and physical functioning by NYHA class and SF-36 physical functioning score. Activity was reported as mean time active/day across 30-days prior to enrolment (30-day PA). Multivariable regression methods were utilised to estimate associations between PA and frailty/functioning (OR = odds ratio, β = beta coefficient, CI = confidence intervals).

**Results:**

Data were available for 140 participants (median age 73, 70.7% male). Median 30-day PA across the analysis cohort was 134.9 min/day (IQR 60.8–195.9). PA was not significantly associated with Fried frailty status on multivariate analysis, however was associated with gait speed (β = 0.04, 95% CI 0.01–0.07, *p* = 0.01) and measures of physical functioning (NYHA class: OR 0.73, 95% CI 0.57–0.92, *p* = 0.01, SF-36 physical functioning: β = 4.60, 95% CI 1.38–7.83, *p* = 0.005).

**Conclusions:**

PA from cardiac devices was associated with physical functioning and gait speed. This highlights the importance of reviewing remote monitoring PA data to identify patients who could benefit from existing interventions. Further research should investigate how to embed this into clinical pathways.

**Supplementary Information:**

The online version contains supplementary material available at 10.1186/s12877-024-05083-1.

## Background

Traditional frailty and physical functioning assessments can be subjective and time consuming to undertake in clinical practice. Sensor technologies can assist by providing objective data on specific physical parameters such as movement, gait and balance, but have been limited by practical constrains such as battery life and compliance [1–3]. Physical activity, as measured by embedded accelerometery within implanted cardiac devices (pacemakers, defibrillators and cardiac resynchronisation therapy devices) offers a novel, passively measured biomarker for older people at risk of frailty without additional burden of assessment. This data commonly contributes to multivariable risk stratification scores used for heart failure stability monitoring [4–7], and as a stand-alone metric has been show to predict heart failure hospitalisation and death [8, 9]. However, utility to assist in the evaluation of frailty or physical functioning in an older population has not been well explored. This is important as older people with frailty, heart disease and low activity represent a key target for multi-modal interventions such as comprehensive geriatric assessment and tailored rehabilitation [10, 11].

Prior research has demonstrated an association between device measured physical activity post-implant and both NYHA (New York Heart Association) functional class [12, 13] and the 6-minute walk endurance test [14] across cardiac resynchronisation therapy (CRT) and implanted cardiac defibrillator (ICD) devices. Only one prior study has investigated association with frailty (limited to non-CRT devices), finding a strong association with gait speed, Timed Up and Go test, and Osteoporotic Fractures instrument measured frailty [15]. Despite these findings, and an increasing body of literature to highlight the importance of identifying and managing frailty in this population [16], activity data from cardiac device remote monitoring is not routinely reviewed in clinical practice. To better explore how this data could be used to enhance clinical care, this study aimed to map out the association between device-measured physical activity with measures of frailty and physical functioning in an ambulatory UK cohort.

## Methods

The PATTErn study (A study of Physical Activity paTTerns and major health Events in older people with implantable cardiac devices, NCT03544424) was a single-site cross-sectional study. Participants were recruited from Manchester Heart Centre, UK between October 2018 and November 2020. Collection of physical activity data was restricted to pre-COVID-19 due to impact of pandemic measures on usual activity levels [17].

Participants were included if they were aged 60 years or over and had a functioning cardiac device in situ compatible with Medtronic, Inc CareLink® remote monitoring application. Participants were excluded if they were unable to walk in upright position or give written informed consent in the English language. At least 6 months of continuous physical activity data prior to enrolment was required for inclusion into the analysis cohort.

### Data collection and definitions

Physical activity data were downloaded directly from in situ cardiac devices (via Bluetooth receiver). Hospitalisation data were obtained from NHS England which collects details of all National Health Service hospital admissions and recorded deaths across the United Kingdom (UK) [18]. All other data were collected from face-to-face assessments or electronic patient records at time of enrolment.

Physical activity was measured by accelerometers embedded within cardiac devices. Each minute was logged as “active” or “sedentary” based on a threshold equivalent to approximately 70 steps [19]. Activity data was processed into mean monthly activity for analyses, as daily variability was not considered clinically significant. The primary metric used for this analysis was “30-day activity”, defined as average physical activity over the 30-days preceding enrolment for a given participant (minutes per day, “mins/day”). This time-frame was selected in line with remote monitoring metrics already in clinical use [20]. Thirty-day activity was compared to activity across the entire monitoring window (six to fourteen months) to identify participants where 30-day activity differed from longer-term behaviour (considered a difference of greater than 60 min), although data from these participants were not excluded. Seasonal variability was also reported in the results.

The main frailty metric used for this study was the Fried criteria (Table 1) by face-to-face assessment at time of enrolment. A Fried score ≥ 3 signified frailty, 1–2 “pre- frail”, and 0 “no frailty” based on a phenotypic approach to frailty [21]. Gait speed was considered both a component, and a stand-alone measure of frailty, recorded as a continuous variable and categorised as “not slow” >0.8 m/second (m/s) “slow”: 0.65–0.8 m/s, “very slow”: 0.5–0.64 m/s and “extremely slow or unable to perform”: <0.50 m/s [22–25]. Self-reported physical activity was measured by the Rapid Assessment of Physical Activity (RAPA) questionnaire (aerobic score 0–7). For the Fried frailty score, low self-reported activity was defined as a RAPA aerobic score < 6. For this paper, self-reported activity was additionally grouped into; active (score 6+), under-active (2–5) and sedentary (< 2). Note this score measures activity in accordance to recommended exercise levels rather than pure activity. A timeframe of 12-months was selected to record a recent history of unplanned hospitalisation, felt to represent a clinically meaningful metric of emergency healthcare utilisation in an older cardiac cohort.

**Table 1:** Fried Frailty Criteria

Domain	Measure	Assessment	Interpretation
Weight loss	Weight	(1) Single question: have you lost more than …kg (10% body weight) in the last 4 years? (2) Height and weight	1 point in case of either: (1) Positive response to single question (2) BMI < 18.5 kg/m^2^ [26]
Muscle weakness	Grip strength [27, 28]	Jamar J00105 hydraulic hand dynamometer Southampton protocol [28]	1 point if in lowest 20% of Cardiovascular Health Study population^1^
Exhaustion	Self-reported	(1) How often in the past week did you feel like everything you did was an effort? (2) How often in the past week did you feel like you could not get going? Responses: often [i.e., ≥ 3 days], not often [i.e., 0–2 days]	1 point in case of ‘often’ response to either question
Slow gait speed	Gait speed [22–24]	Participant asked to walk 5 m at normal walking speed.	1 point if gait speed < 0.8 m/s, or unable to perform
Low physical activity	Self-reported	RAPA questionnaire [29]	One point if RAPA Aerobic score < 6

^1^As described in appendix of Fried et al., 2001 [21]. *Abbreviations*: BMI = body mass index, RAPA = the Rapid Assessment of Physical Activity

Physical functioning was measured by New York Heart Association Functional Classification (NYHA class) and the physical functioning component of the SF-36 (Medical Outcomes Study Questionnaire Short Form 36 Health Survey) [30–32]. Activities of daily living included both basic and instrumental activities [33]. A history of falls was defined as at least one fall in the last 12-months, and “adapted living” as a house with adaptions (for example ramps, stairlift, grab rails etc.), sheltered or “warden-controlled” accommodation, or residential care. Mobility was taken as how a participant usually walked outside. Quality of life was measured using the mental health component of the SF-36.

### Statistical methods

Data were summarised using the mean with standard deviation (SD) in the case of normally distributed data, otherwise the median with upper and lower quartiles was used. All reference to “average” refers to the mean value and a p-value < 0.05 was considered statistically significant. Small counts (< 5) were supressed in line with data protection guidance [34]. For comparative analysis of categorical variables, the Kruskal-Wallis rank sum test (K-W test) was performed for non-parametric data, with pair-wise comparisons using the Wilcoxon test with Bonferroni correction. For continuous variables, Spearman correlation was used. Outliers were defined as data points 1.5 times IQR above the third quartile or below the first quartile.

Complete case multivariable regression was used to estimate associations between device measured physical activity and measures of frailty and physical functioning, adjusted for age, gender, body mass index, heart failure, device type and unplanned hospitalisation in the past 12-months (covariates selected on prior literature and clinical relevance [35, 36]). Physical activity was converted to hours per day to ease interpretation without loss of data. Frailty, NYHA and SF36 scores were considered “outcome variables”, assessed on date on enrolment. Linear regression was used to estimate associations between 30-day activity and SF36 scores. For frailty status and NYHA class, odds ratios (OR) were estimated using ordinal logistic regression. For highly skewed 30-day activity data, a sensitivity analysis was performed using groupings (< 1 h/day, 1–2 h/day, 2–3 h/day, 3 + hours/day) in addition to ordinal regression. Full results of multivariable analyses performed are available in the supplementary material.

All data selection, cleaning and formatting activities were performed using software “R” version 4.1.3 [37].

## Results

### Study population

Overall, 183 participants were recruited into the PATTErn study, with 140 participants in the analysis cohort (43 excluded due to unobtainable device data). The demographics of the analysis cohort are provided in Table 2.

**Table 2:** Analysis cohort demographics

	Analysis cohort (*n* = 140)	Missing data (*n*)
Age	73.0 [12.0]	0
Male	99 (70.7%)	0
BMI	28.8 (5.2)	2
Device		0
CRTD	53 (37.9%)	-
CRTP	38 (27.1%)	-
ICD	27 (19.3%)	-
Pacemaker	22 (15.7%)	-
Heart failure	100 (71.4%)	0
NYHA class		2
I	61 (44.2%)	-
II	56 (40.6%)	-
III/IV	21 (15.2%)	-
Atrial fibrillation/flutter	83 (59.3%)	0
Ischaemic heart disease	79 (56.4%)	0
Diabetes	35 (25.0%)	0
Chronic kidney disease stage 3+	64 (45.7%)	0
COPD	21 (15%)	0
Frailty status		0
Not frail	50 (35.7%)	-
Pre frail	74 (52.9%)	-
Frail	16 (11.4%)	-
Gait speed (m/s)	1.0 (0.3)	3
Slow gait speed (< 0.8 m/s)	33 (23.6%)	-
Low grip strength	35 (25.0%)	0
Self-reported physical activity		3
Sedentary	12 (8.6%)	-
Under-active	92 (65.7%)	-
Active	33 (23.6%)	-
Exhaustion	48 (34.3%)	0
Weight loss	26 (18.6%)	0
SF-36 Physical functioning^1^	75.0 [55.0]	2
SF-36 Mental health^1^	78.1 (16.4)	2
Assistance with ADLs	38 (27.1%)	5
History of falls^2^	23 (16.4%)	2
Adapted living^3^	29 (20.7%)	0
Has a carer^4^	10 (7.1%)	2
Walking aids^5^	38 (27.1%)	1
> 2 GP visits^6^	42 (30%)	2
Unplanned hospitalisation in the past 12-months ^7^	12 (8.6%)	0

^1^A proportional 0-100 scale ranging from 0 (worst) to 100 (best), ^2^self-reported falls in last 12-months, ^3^defined as a house with adaptions, sheltered accommodation or residential care, ^4^carer defined as “anyone who helps to look after you in your day-to-day”, excluding care home staff, ^5^how participant usually walks outside, ^6^unplanned visits to General Practitioner, ^7^nunplanned hospitalisation in the 12 months prior to recruitment

*Abbreviations*: BMI = body mass index, CRT = cardiac resynchronisation therapy device (-P with pacemaker, -D with defibrillator), ICD = implanted defibrillator, NYHA = New York Heart Association functional class, COPD = chronic obstructive pulmonary disease, ADL = activities of daily living, SF-36 = Medical Outcomes Study Questionnaire Short Form 36 Health Survey

### Measured physical activity

Days of available activity data per participant ranged from 206 to 425, with 20 participants recording < 425 days of activity data. Daily activity ranged from 0 to 688.0 min/day. Thirty-day activity data was skewed towards zero, with fourteen participants recording less than 30 min/day. Median 30-day activity across the analysis cohort was 134.9 min/day (IQR 60.8–195.9). There were four cases where thirty-day activity differed from the participant’s mean activity across all monitored time by greater than 60 min. On visual inspection of the participant-level trended data in these cases, mean monthly activity was highly variable across the monitoring period (not always unidirectional).

Median daily activity tended to be lower in the winter months (spring: 137.0 min/day IQR 65.0–222.0, summer: 138.0 min/day IQR 63.0–221.0, autumn: 132.0 min/day IQR 58.0–214.0 and winter: 126.0 min/day, IQR 56.5–205.0).

### Physical activity and frailty

**Fig. 1:**
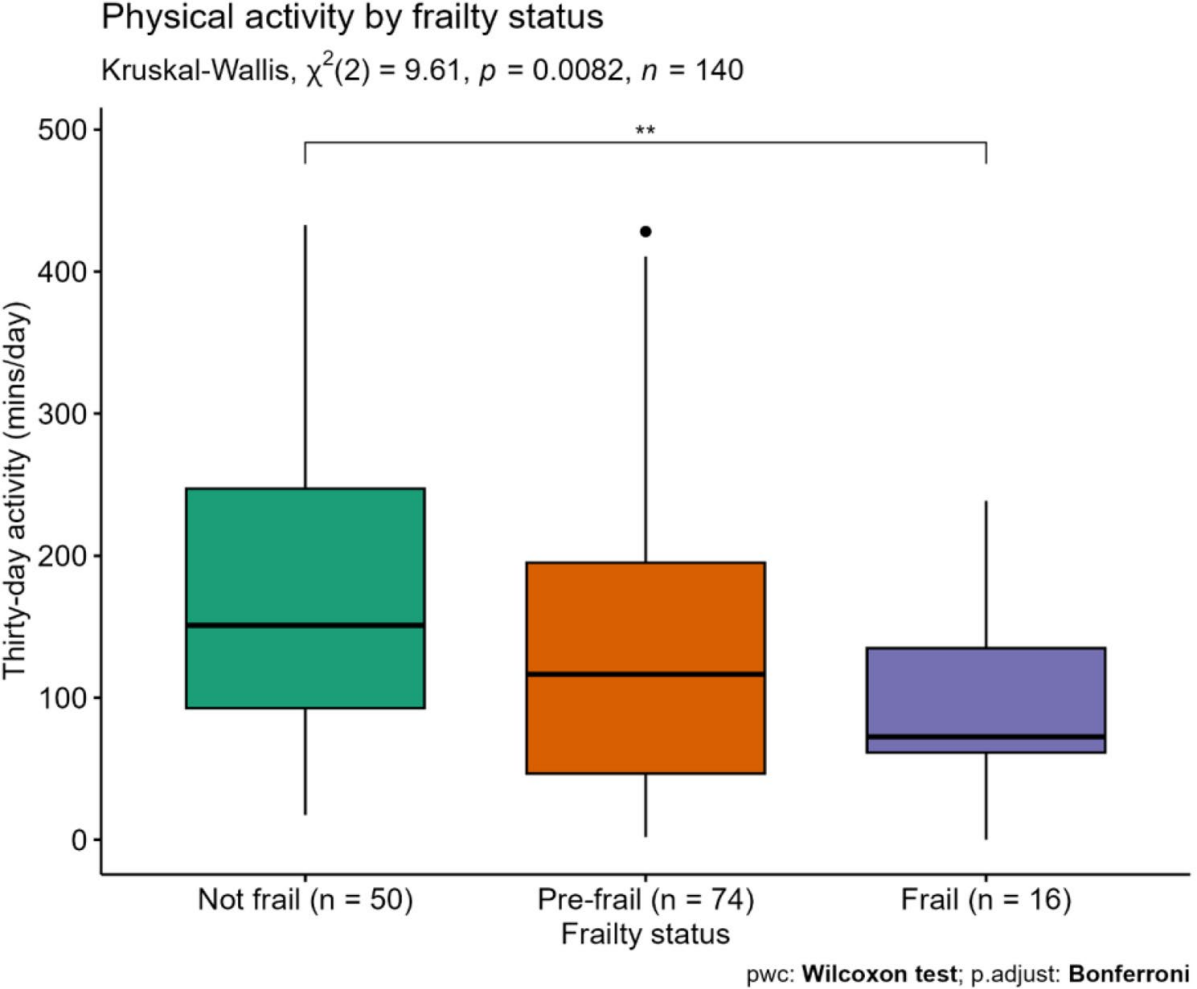
Thirty-day physical activity by frailty status. Abbreviations pwc = pairwise comparison, p.adjust = adjusted p-value

Median thirty-day activity across frailty groups as measured by the Fried criteria were as follows; non-frail participants: 151.0 min/day IQR 92.6–247.1, pre-frail: 116.6 min/day IQR 46.6–195.1, frail: 72.5 min/day IQR 61.4–134.9. Activity was significantly different across the frailty groups with an incremental decrease in activity across frailty groups (Fig. 1). There were two significant outliers in the pre-frail group, and one in the not frail group, all with very high thirty-day activity. Of note, of the two outliers with pre-frailty, neither reported low activity and both had normal gait speed.

When adjusting for demographic and clinical characteristics, 30-day physical activity was not independently associated with frailty status (OR 0.82, 95% CI 0.65–1.03, *p* = 0.091, see Table 3). Similar results were seen when the grouped 30-day activity variable was used (*see Supplementary Material*).

**Table 3:** Thirty-day activity and frailty status: Ordinal logistic regression analysis (*n* = 138)

Explanatory variable^1^	Odds Ratio	95% CI	*p*-value
30-day physical activity^2^	0.82	0.65–1.03	0.091
Age	1.04	0.99–1.09	0.090
Gender (female)	1.51	0.73–3.19	0.269
Body mass index	1.01	0.94–1.09	0.740
Heart failure	1.45	0.62–3.41	0.395
Device (CRT versus non-CRT)	1.06	0.49–2.30	0.891
Unplanned hospitalisation in the past 12-months	2.35	0.71–7.91	0.159

^1^2 cases missing body mass index data^2^average daily activity in 30-days prior to enrolment (hours/day)

*Abbreviations*: CI = confidence interval, CRT = cardiac resynchronisation therapy

**Fig. 2:**
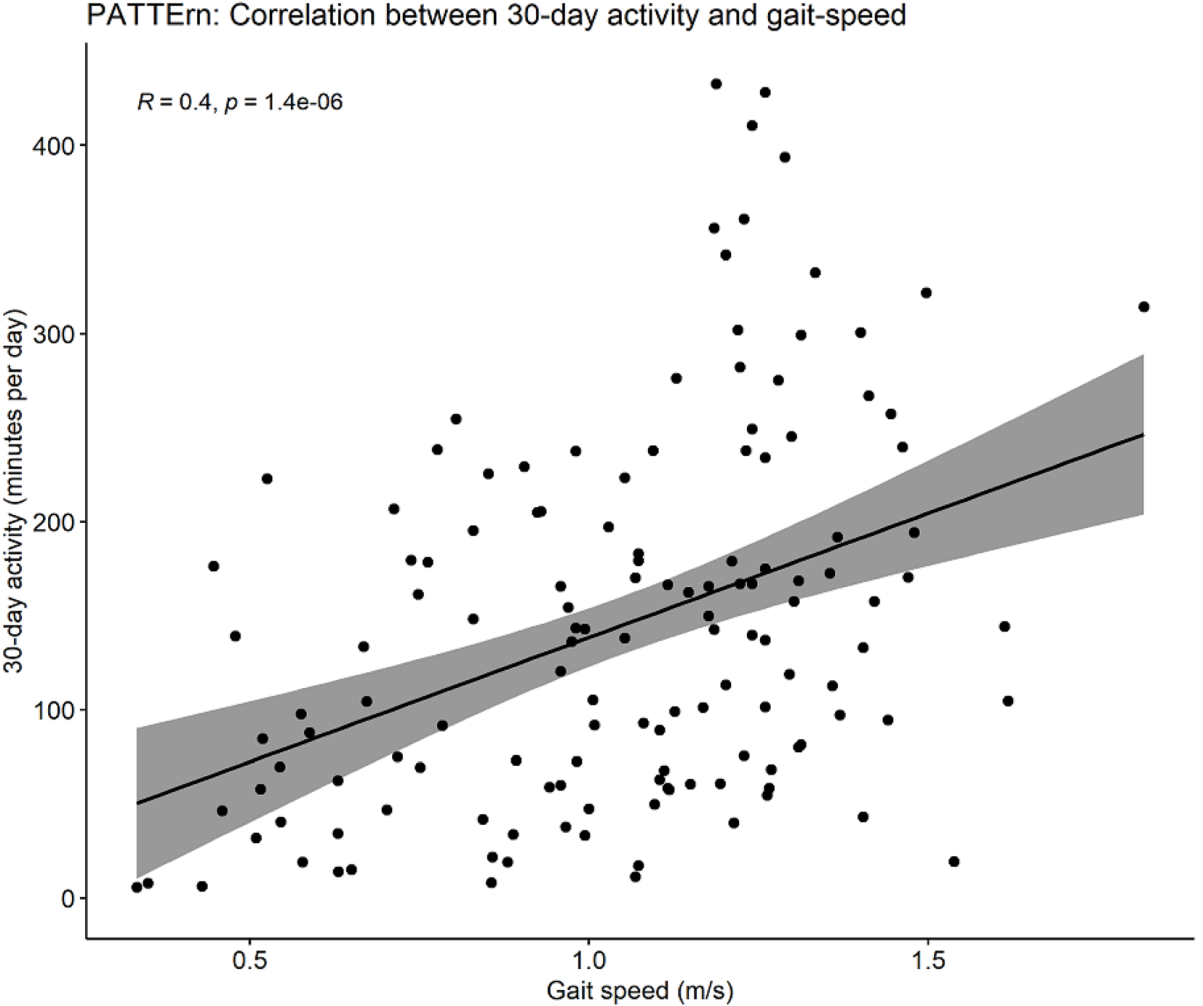
Pattern study Correlation between 30-day activity by gait speed

Physical activity was greater in faster gait speed categories. Median 30-day activity was 148.3 min/day IQR 74.4–227.4 for participants with normal gait speed (*n* = 107), 118.9 min/day IQR 73.8–178.8 for slow gait speed (*n* = 12), 60.3 min/day IQR 33.8–85.5 for very slow gait speed (*n* = 12) and 8.1 min/day IQR 5.7–65.6 for extremely slow or unable to perform participants (*n* = 9). Gait speed data was negatively skewed, and activity data positively skewed. Three patients had no gait speed measurement as they were unable to perform the walk test. There was a moderate linear correlation between gait speed and 30-day physical activity and gait speed (Spearman correlation coefficient = 0.40, *p* < 0.001, *n* = 137, see Fig. 2*).*

After adjustment for demographic and clinical characteristics through linear regression analysis, every additional hour of daily physical activity in the 30 days prior to enrolment was associated with a 0.04 m/s increase in measured gait speed (95% CI 0.01–0.07; *p* = 0.01; *full results available in Supplementary material*).

**Fig. 3:**
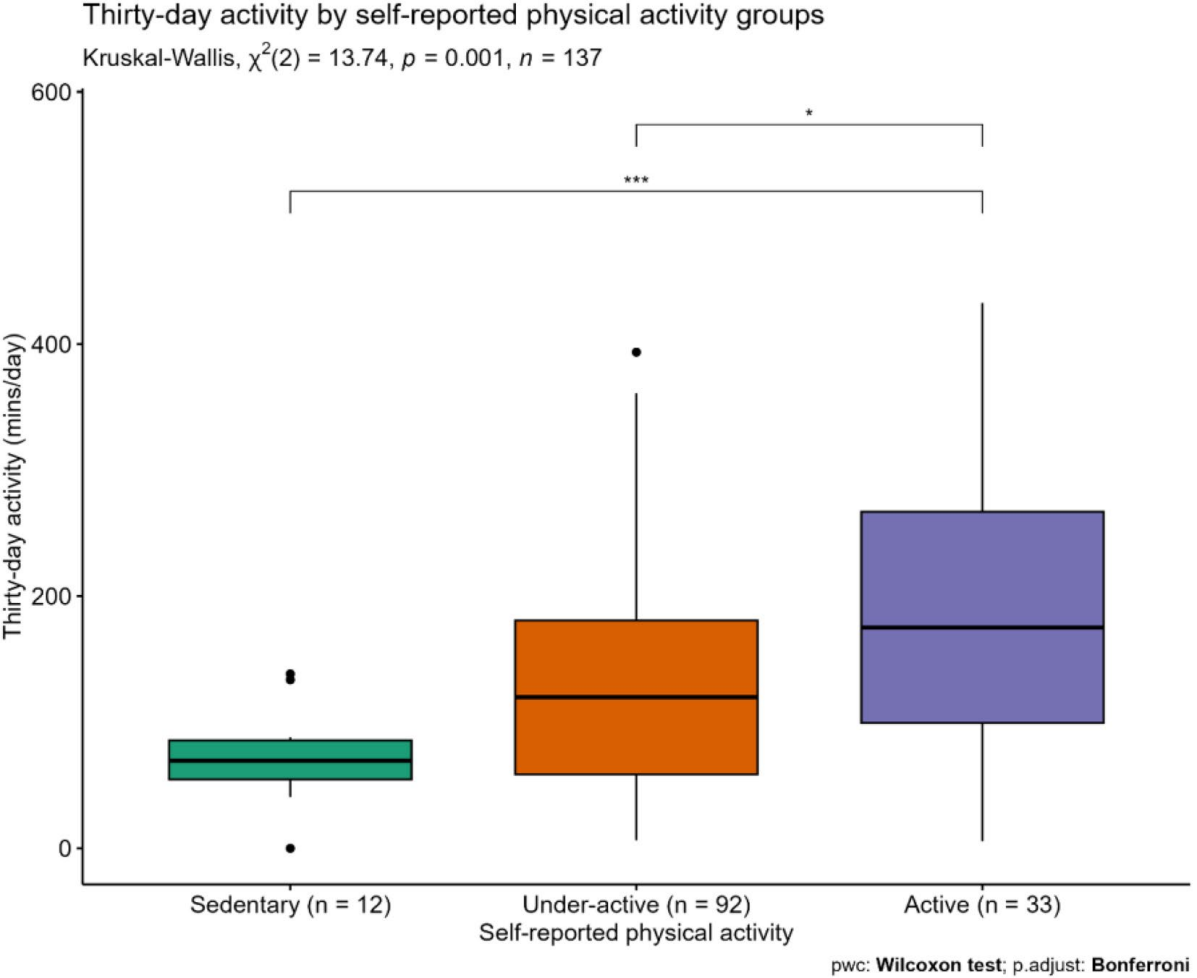
Thirty-day activity by self -reported physical activity groups. Note there were four outliers (sedentary group *n* = 3, under-active *n* = 1)

Device-measured physical activity was different between self-reported physical activity groups; active: 175.0 min/day IQR 99.2–267.0; under-active: 119.7 min/day IQR 58.8–180.6; sedentary: 69.6 min/day IQR 54.8–85.5; *p* = 0.001, see Fig. 3 (note three cases with missing data).

Median thirty-day activity was similar across groups with different grip strength (by Fried frailty assessment criteria). Thirty-day activity was 136.4 min/day IQR 63.0–205.0 in participants with low grip strength, compared to 133.5 min/day IQR 58.7–173.4 for participants normal/high grip strength (K-W test, *p* = 0.4). For patients reporting exhaustion, 30-day activity was significantly lower (median 30-day activity 77.6 min/day IQR 46.9–168.8 versus 146.3 min/day IQR 80.1–227.6, *p* = 0.005 on K-W test). For patients reporting weight loss, 30-day activity was not significantly different, with a wide spread of activity recorded (median 30-day activity 91.0 min/day IQR 41.3–202.3 versus 138.7 min/day IQR 66.1–194.5, *p* = 0.3 on K-W test).

### Physical activity and measures of physical functioning

**Fig. 4:**
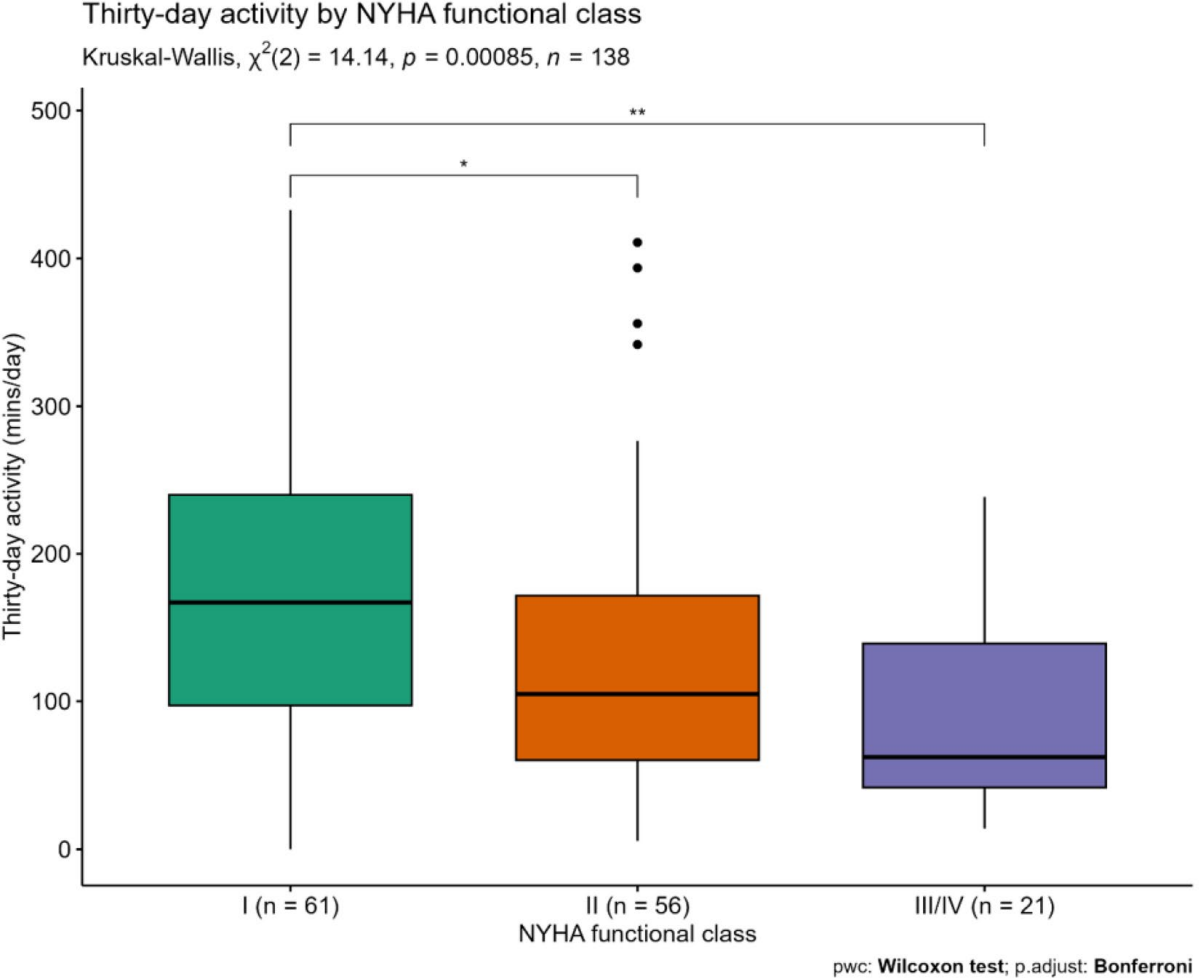
30-day physical activity by NYHA functional class. Of note there were four “high” outliers in the NYHA class II group, all with normal gait speed and either not frail or pre-frail Abbreviations pwc = pairwise comparison, p.adjust = adjusted p-value

NYHA data were available from 138 (98.7%) participants. Participants with poorer NYHA score tended to have lower 30-day activity as shown in Fig. 4 (class I: 166.9 min/day IQR 97.3–240.0; class II: 104.9 min/day IQR 60.4–171.7; class III/IV: 62.5 min/day IQR 42.0–139.2). After adjusting for age, sex, BMI, device type and unplanned hospitalisation in the past 12-months, 30-day activity was still associated with NYHA class at enrolment (OR 0.73, 95% CI 0.57–0.92, *p* = 0.01, *full results available in Supplementary material*).

SF-36 physical functioning scores were available for 138 (98.7%) participants. After adjusting for covariates above with the addition of heart failure, there was a significant association between 30-day activity and SF-36 physical functioning scores (β = 4.60, 95% CI 1.38–7.83, *p* = 0.005, *full results available in Supplementary material*).

## Discussion

The key finding of this analysis is that physical activity detected by cardiac devices did not strongly associate with phenotypic frailty, however did associate with gait speed and physical functioning.

Although results showed physical activity was lower in participants with frailty, association on multivariable analysis was not significant. This may have been a consequence of the frailty measurement instrument selected, with participants defined as “pre-frail” due to weight loss or poor grip strength rather than slow gait speed or low self-reported aerobic exercise were likely to represent outliers in the analysis. Only one prior study has compared cardiac device-measured activity with frailty (Kramer et al., 2017 [15]). In this study, the Study of Osteoporotic Fractures instrument was used, reporting similar results. Future studies should consider additional index-based “cumulative deficit” approaches to compare results to better explore the relationship between activity and different conceptual models of frailty. Readers should also bear in mind methodological considerations such as a small frailty group (*n* = 16), and limitations of ordinal regression.

Both physicalactivity and gait speed are influenced by physical mobility. Kramer et al., 2017 also measured gait speed, finding a smaller magnitude of association which was not significant on multi-variable analysis. Of note, the cohort recruited by Kramer et al., 2017 was significantly younger with less severe heart failure and faster gait speed, therefore differences in results may reflect cohort composition. Physical endurance was not measured by this study, however in the literature Vegh et al., 2014 reported significant correlation between activity and six-minute walking test (6 months post-implant, *r* = 0.392, *p* < 0.01). Although the association between physical activity and NYHA has been reported before, this metric is heavily focused on heart failure symptom severity. Our study is the first to report on the association between cardiac device monitored activity and self-reported physical functioning using a comprehensive measurement tool.

The main immediate clinical implication of this study is to advocate the use of remote monitoring physical activity data to complement clinical assessment. Although diagnostic testing was not applied to this dataset due to lack of accepted thresholds and small sample size, it is reasonable to utilise physical activity data from devices to aid identification of patients who may benefit from rehabilitation, lifestyle support strategies, comprehensive geriatric assessment or palliative care. This would be in line with pre-existing clinical guidelines which strongly supporting the referral of people with heart failure and sedentary activity to exercise and rehabilitation schemes [38–40]. Existing evidence would suggest sedentary behaviour interventions can, in addition to improving cardiovascular health, delay frailty progression [41, 42]. There is also a recognised clinical need to better identify patients on a trajectory into end of life care [43]. Further research is required to ascertain if such strategies would be effective. The use of cardiac device physical activity data as a stand-alone screening tool for frailty or poor physical functioning is not supported by this study.

Physical activity measured directly by cardiac devices represents a unique metric which provides additional clinical insights into patient behaviour. Its place alongside traditional measures of frailty and physical functioning has not yet been established, however this research (alongside the published literature) would suggest value as either a stand-alone metric, or a component measure of existing clinical frailty scores. This could be achieved by replacing or complementing existing physical activity questionnaires. This would need to be tested in prospective studies where key outcome such as mortality, hospitalisation and institutionalisation are captured. The role of physical activity to predict worsening frailty should also be explored, as this would drive a different spectrum of interventions. Additional derived metrics from physical activity monitoring such as trended data may also provide predictive value, representing a research area in need of development.

## Limitations

As reported, for a small number of patients 30-day activity was not representative of “usual” activity levels. Most self-report and wearable studies have used a 7-day monitoring period, these methods were limited by recall bias and device compliance, highlighting one of the key advantages of using implanted technologies [44, 45]. Thirty-days was felt to more accurately represent “normal” activity and provide a meaningful metric for clinical use. More extended timeframes were considered (in particular to mitigate for seasonality), however this would risk capturing trends due to natural progression of disease (note approximately 20% of patients die within a year of diagnosis of heart failure in the UK) [46]. Ultimately, reduced activity for as little as two weeks has been shown to have delirious cardiorespiratory and metabolic effects, therefore remains relevant for physical functioning [47].

Small sample size limited statistical power. Data for 43 participants was lost due to unobtainable device data, and additional enrolment was not possible due to the outbreak of COVID-19. Ordinal regression assumes equal difference between outcome categories (e.g. not frail, pre-frail, frail) which was unlikely to be the case. A sensitivity analysis was included to partially address this issue, however future studies should consider measuring frailty using a spectrum of approaches to provide additional certainty, for example a frailty index.

Inclusion criteria was restricted to patients with devices compatible with Medtronic technology, with limited enrolment of patients with pacemakers and exclusion of cardiac monitoring systems. The remote technology used for this project was designed for heart failure remote monitoring, therefore devices implanted primarily to monitor/manage arrythmias were often incompatible. Accelerometer data for this data was uni-axial, therefore may not capture all methods of exercise.

### Key clinical implications

1. Practitioners can utilise remote monitoring data form cardiac devices to aid comprehensive geriatric assessment for older people with heart failure.
2. Existing national guidance (UK) would support referral of patients with heart failure and low activity levels to exercise or rehabilitation schemes.
3. The use of cardiac device physical activity data as a stand-alone screening tool for frailty or poor physical functioning is not supported by this study.

## Conclusion

Physical activity from cardiac devices is associated with physical functioning, and some components of clinical frailty. Practitioners should consider routinely reviewing physical activity data from remote monitoring to identify patients who may benefit from exercise interventions.

### Electronic supplementary material

Below is the link to the electronic supplementary material.

Supplementary Material 1

## Data Availability

The data that support the findings of this study are not openly available due to reasons of sensitivity, however anonymised data will be made available from the corresponding author upon reasonable request.
