# Supplementary material for: Remotely monitored physical activity from older people with cardiac devices associates with physical functioning
Source: BMC Geriatr. 2024 Jun 17;24:526. doi: 10.1186/s12877-024-05083-1 (PMC11184810; doi:10.1186/s12877-024-05083-1)
Supplement: Supplementary file 1 — Supplementary Material 1 [file 12877_2024_5083_MOESM1_ESM.docx]

**Supplementary Table 1: Sensitivity analysis: grouped thirty-day activity and frailty status: Ordinal logistic regression analysis (n = 138)**

| Explanatory variable^1^ | Odds Ratio | 95% CI | p-value |
| --- | --- | --- | --- |
| Grouped 30-day physical activity^2^ | 0.72 | 0.46 – 1.11 | 0.134 |
| Age | 1.04 | 0.99 – 1.10 | 0.693 |
| Gender (female) | 1.45 | 0.70 – 3.04 | 0.321 |
| Body mass index | 1.01 | 0.94 – 1.08 | 0.749 |
| Heart failure | 1.58 | 0.70 – 3.64 | 0.275 |
| Device (CRT versus non-CRT) | 1.02 | 0.47 – 2.23 | 0.956 |
| Unplanned hospitalisation in the past 12-months | 2.50 | 0.76 – 8.41 | 0.133 |
| *^1^2 cases missing body mass index data (note in addition 3 cases missing gait speed data), ^2^groupings <1 hour/day, 1-2 hours/day, 2+ hours/day*  *Abbreviations: CI = confidence interval, CRT = cardiac resynchronisation therapy* | | | |

**Supplementary Table 2: Physical activity and gait speed: multiple linear regression analysis (n = 136)**

| Explanatory variable^1^ | Coefficient (β) | 95% CI | p-value |
| --- | --- | --- | --- |
| 30-day physical activity | 0.04 | 0.01 - 0.07 | 0.013* |
| Age | -0.02 | -0.02 - -0.01 | <0.001* |
| Gender (Female) | -0.07 | -0.16 - 0.02 | 0.143 |
| Body mass index | -0.01 | -0.02 -0.00 | 0.012* |
| Heart failure | -0.09 | -0.20 - 0.02 | 0.113 |
| Device (CRT versus non-CRT) | -0.03 | -0.13 - 0.06 | 0.503 |
| Unplanned hospitalisation in the past 12-months | -0.18 | -0.33 - -0.03 | 0.016* |
| *^1^Three participant excluded from analysis due to missing gait speed (unable to perform) and one due to missing body mass index.*  **p <0.005*  *Abbreviations: CI = confidence interval, CRT = cardiac resynchronisation therapy* | | | |

**Supplementary Table 3: 30-day activity and NYHA class: ordinal logistic regression analysis (n = 138)**

| Explanatory variable^1^ | Odds ratio | 95% CI | p-value |
| --- | --- | --- | --- |
| 30-day physical activity | 0.71 | 0.56 – 0.88 | 0.002* |
| Age | 1.00 | 0.95 – 1.04 | 0.84 |
| Gender (female) | 1.42 | 0.6 – 2.95 | 0.35 |
| Body mass index | 1.05 | 0.98 – 1.12 | 0.20 |
| Device (CRT versus non-CRT) | 1.40 | 0.68 – 2.91 | 0.37 |
| Unplanned hospitalisation in the past 12-months | 1.66 | 0.55 – 5.00 | 0.36 |
| *^1^Two participants excluded due to missing NYHA class, and one excluded due to missing body mass index*  **p <0.005*  *Abbreviations: NYHA = New York Heart Association functional classification, CI = confidence interval, CRT = cardiac resynchronisation therapy* | | | |

**Supplementary Table 4: 30-day activity and SF-36 physical functioning: multiple linear regression analysis**

| Explanatory variable^1^ | Coefficient (β) | 95% CI | p- value |
| --- | --- | --- | --- |
| 30-day activity | 4.60 | 1.38 - 7.83 | 0.005* |
| Age | -0.47 | -1.14 - 0.20 | 0.171 |
| Gender (Female) | -7.43 | -17.91 – 3.06 | 0.164 |
| Body mass index | -0.89 | -1.87 - 0.09 | 0.07 |
| Heart failure | -17.28 | -29.40 - -5.17 | 0.006* |
| Device (CRT versus non-CRT) | -1.11 | -11.96 – 9.74 | 0.840 |
| Unplanned hospitalisation in the past 12-months | -13.03 | -30.39 – 4.34 | 0.140 |
| *^1^Two cases excluded due to missing SF-36 physical functioning score, and one case excluded due to missing body mass index.*  **p <0.005*  *Abbreviations: CI = confidence interval, CRT = cardiac resynchronisation therapy* | | | |
